# Menstrual cycle patterns in primary care: a structured approach using primary ovarian insufficiency as a sentinel condition

**DOI:** 10.3389/fmed.2026.1795149

**Published:** 2026-06-02

**Authors:** Lawrence Merle Nelson

**Affiliations:** Mary Elizabeth Conover Foundation Inc., McLean, VA, United States

**Keywords:** amenorrhea, clinical triage, early recognition, longitudinal care, menstrual cycle as a vital sign, menstrual cycle patterns, primary care, primary ovarian insufficiency

## Abstract

Primary Ovarian Insufficiency, a condition characterized by early loss of ovarian function, illustrates the consequences of failing to act on menstrual cycle changes as early clinical signals. Menstrual disturbance is often the first presenting feature, yet diagnosis is frequently delayed despite repeated clinical encounters. For primary care clinicians, a disrupted menstrual pattern in an adolescent or young adult should prompt consideration of whether Primary Ovarian Insufficiency could be present in an early stage. This Perspective presents a structured approach to interpreting menstrual cycle patterns, an increasingly recognized vital sign, as actionable clinical signals in primary care. Using Primary Ovarian Insufficiency as a sentinel condition, menstrual changes are framed as prompts for timely decisions to evaluate, refer, or follow, based on pattern, persistence, progression, and associated clinical features. This approach integrates standard diagnostic pathways with contextual clinical information, including metabolic status, stress physiology, and environmental exposures, without adding to the burden of routine care. Extending beyond Primary Ovarian Insufficiency, the framework applies across the lifespan, including midlife hormonal transition, where menstrual changes similarly reflect underlying physiological change and are often under-interpreted. Making explicit how menstrual patterns inform clinical decision-making supports earlier recognition of change, more consistent triage, and improved alignment between patient presentation and clinical response. Strengthening the interpretation of menstrual cycle patterns in primary care, particularly within longitudinal, continuous care relationships, offers a practical opportunity to improve the timing and quality of care by leveraging information already present in routine clinical encounters.

## Introduction

Primary Ovarian Insufficiency illustrates the clinical consequences of failing to recognize menstrual cycle changes as early signals of reproductive endocrine dysfunction. Menstrual disturbance, including irregular cycles or secondary amenorrhea, is often the first presenting feature, yet diagnosis is frequently delayed despite repeated clinical encounters (1–3). This delay is clinically significant because Primary Ovarian Insufficiency is not limited to fertility implications; it is associated with systemic health consequences, including reduced bone mineral density, increased cardiovascular risk, and adverse psychosocial outcomes (4–6).

Menstrual cycle patterns reflect integrated hypothalamic–pituitary–ovarian axis activity rather than isolated ovarian function. They provide routinely available clinical information and have been increasingly recognized as a vital sign in women’s health (7–9). In primary care, however, menstrual changes are often documented but not consistently interpreted as actionable clinical signals, particularly when they occur outside acute or severe presentations. Primary Ovarian Insufficiency serves as a sentinel condition through which this gap can be examined, as early menstrual changes are commonly present before diagnosis but may be normalized, attributed to developmental or contextual factors, or reassessed only after progression has occurred (2, 10).

A structured approach to menstrual cycle interpretation requires more than recording whether menses are present or absent. It requires attention to predefined clinical features, including cycle frequency, regularity, duration, volume, amenorrhea, persistence over time, progression, and associated symptoms such as features of estradiol deficiency. Standardized terminology for menstrual bleeding patterns provides the foundation for such interpretation, including criteria related to frequency, regularity, duration, and volume (11, 12). These elements help determine whether a menstrual pattern should prompt diagnostic evaluation, referral, or planned follow-up. Such an approach is especially relevant after menarche, when normal developmental variability may coexist with early signs of endocrine, genetic, or gynecologic conditions, including Primary Ovarian Insufficiency (8, 13).

For primary care clinicians seeking a broader diagnostic approach to amenorrhea, Klein et al. provide a practical review of evaluation and management in a primary care context (14). The present Perspective builds on that clinical foundation by focusing specifically on how menstrual cycle patterns can be interpreted as early signals for Primary Ovarian Insufficiency and used to guide decisions to evaluate, refer, or follow over time.

**Figure 1:**
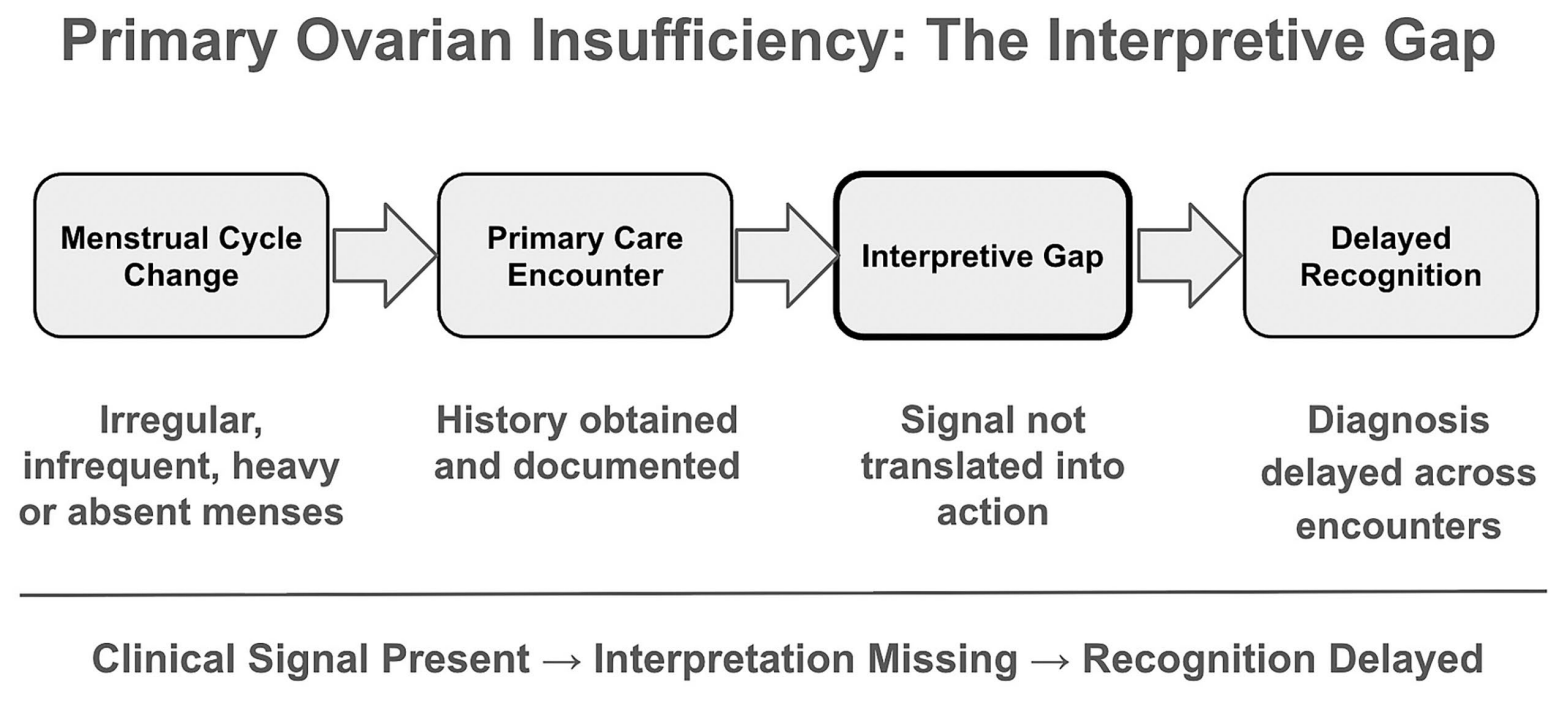
Primary ovarian insufficiency: the interpretive gap. Menstrual cycle changes may precede recognition of primary ovarian insufficiency but remain under-interpreted in routine care. When menstrual history is documented without being translated into clinical action, recognition may be delayed.

Primary care provides a setting in which this interpretive gap can be addressed. Continuity of care supports recognition of patterns over time and allows menstrual changes to be interpreted within a broader clinical context, including metabolic status, stress physiology, medication exposure, and environmental factors (15–17). Environmental exposures, including endocrine-disrupting chemicals, are increasingly recognized as relevant to reproductive endocrine health and should be considered within the broader context rather than as isolated explanations for menstrual disturbances (18–20). At the same time, interpretation must remain efficient and aligned with existing workflows, given time constraints within routine clinical encounters (21). The aim is not to add a separate diagnostic burden, but to shift menstrual history from passive documentation to structured clinical reasoning. This interpretive gap is summarized in Figure 1.

This Perspective presents a structured approach to interpreting menstrual cycle patterns in primary care using Primary Ovarian Insufficiency as a sentinel condition. The framework draws on clinical principles and on methodological concepts commonly emphasized in experimental models, including standardized timing of observations, predefined criteria for interpretation, and efforts to reduce interpretive bias (22). Applied in clinical practice, these principles support consistent documentation, explicit action thresholds, and reassessment over time. The following section examines Primary Ovarian Insufficiency as a sentinel condition to illustrate how early menstrual signals can be recognized, interpreted, and acted upon within primary care.

## Primary ovarian insufficiency as a sentinel condition

Primary Ovarian Insufficiency provides a clear example of how early menstrual cycle changes may signal underlying reproductive endocrine dysfunction and how delays in interpretation can affect clinical outcomes. Primary Ovarian Insufficiency is defined as loss of normal ovarian function before age 40, characterized by menstrual disturbance and biochemical evidence of impaired ovarian function, including elevated gonadotropin levels (23, 24). Menstrual disturbance, including irregular cycles, oligomenorrhea, or secondary amenorrhea, is typically the first presenting feature and may precede formal diagnosis by months or years (1–3).

The biological heterogeneity of Primary Ovarian Insufficiency further supports its use as a sentinel condition. Presentations may reflect genetic, autoimmune, iatrogenic, or idiopathic mechanisms, and disease progression may be variable rather than abrupt (13, 22, 24–26). Genetic contributions, including fragile X–associated Primary Ovarian Insufficiency, illustrate that menstrual cycle disturbance may be the visible clinical signal of a broader underlying biological process (22, 26). In this context, the challenge for primary care is not simply to recognize the absence of menses, but to interpret changes in menstrual pattern as a potential early signal requiring structured evaluation.

Delayed recognition has consequences beyond fertility. Primary Ovarian Insufficiency is associated with reduced bone mineral density, cardiovascular risk, evidence of endothelial dysfunction, and adverse psychosocial outcomes (4–6, 27). These systemic implications underscore the importance of timely diagnosis and comprehensive care. They also reinforce why menstrual disturbance should not be treated as an isolated symptom or deferred until prolonged amenorrhea is established.

Patients with Primary Ovarian Insufficiency frequently report fragmented care and multiple clinical encounters before diagnosis, indicating that early signals are often present but underused in decision-making (1–3). Established guidance has long emphasized that menstrual disturbances such as secondary amenorrhea warrant prompt evaluation, and more recent evidence-based guidance continues to emphasize timely recognition and coordinated management (7, 23, 24, 28). The persistent delay, therefore, reflects a gap between available clinical knowledge and consistent implementation in routine care.

Primary Ovarian Insufficiency, therefore, functions as a sentinel condition for structured menstrual cycle interpretation in primary care. It demonstrates how menstrual cycle changes can be clinically meaningful before a diagnosis is made, how normalization or delayed reassessment can postpone care, and how predefined criteria for evaluation and referral may improve timely recognition. Addressing this gap requires clear definitions of menstrual cycle signals and a structured approach to determining when to evaluate, refer, or follow within routine primary care.

## Defining menstrual cycle signals in clinical practice

Interpreting menstrual cycle changes in relation to Primary Ovarian Insufficiency requires consistent terminology and predefined clinical parameters. Menstrual history should not be limited to whether bleeding is present or absent, but should include the core features that define menstrual pattern: cycle frequency, regularity, duration, volume, amenorrhea, persistence over time, progression, and associated clinical features. These elements provide the basis for determining whether a change is likely to represent expected variation, requires diagnostic evaluation, warrants referral, or can be followed with planned reassessment.

Standardized terminology for menstrual bleeding patterns, as established by the International Federation of Gynecology and Obstetrics (FIGO) and related consensus frameworks, provides the foundation for clinical interpretation (11, 12). In reproductive-aged individuals, normal cycle frequency is generally defined as bleeding every 24 to 38 days, with relatively consistent cycle-to-cycle variation. Bleeding lasting more than 8 days, cycles occurring more frequently than every 24 days or less frequently than every 38 days, or increasing variability beyond expected ranges fall outside normal parameters and warrant clinical assessment (12).

Amenorrhea is a particularly important signal in relation to Primary Ovarian Insufficiency. The absence of menses for more than 90 days after previously established cycles is abnormal at any stage after menarche and requires evaluation (7, 8). In adolescents and young adults, this threshold is clinically important because menstrual disturbance may be the presenting feature of Primary Ovarian Insufficiency, even when symptoms are initially attributed to developmental variation, stress, weight change, or other contextual factors.

Abnormal uterine bleeding is defined by deviations in frequency, regularity, duration, or volume. Clinical significance should be determined not only by numerical thresholds but also by the patient’s experience, including bleeding that interferes with daily activities or involves flooding through protection (12, 29). Although abnormal uterine bleeding is not specific for Primary Ovarian Insufficiency, defining the bleeding pattern precisely helps distinguish menstrual cycle disturbance from structural, endocrine, iatrogenic, or other causes that may require separate evaluation.

For Primary Ovarian Insufficiency, the most relevant menstrual signals are those that are persistent, progressive, unexplained, or associated with features suggesting estradiol deficiency or broader endocrine dysfunction. These may include increasing cycle length, oligomenorrhea, secondary amenorrhea, vasomotor symptoms, sleep disturbance, dyspareunia, genitourinary symptoms, or other clinical features that raise concern for estradiol deficiency. When such findings occur in an adolescent or young adult, Primary Ovarian Insufficiency should be considered among the conditions requiring timely evaluation and, when indicated, referral.

This approach adapts methodological principles commonly emphasized in experimental models to clinical practice. Standardized timing means documenting menstrual cycle features consistently across visits rather than relying on isolated recall. Predefined criteria specify which patterns require evaluation, referral, or planned reassessment. Although blinding is not directly applicable to routine clinical care, its underlying purpose, reducing interpretive bias, is relevant. Clinicians should avoid prematurely attributing menstrual disturbance to adolescence, stress, body weight change, or midlife transition without assessing whether the pattern is persistent, progressive, unexplained, or accompanied by concerning clinical features.

Taken together, these definitions establish the clinical signals that structure subsequent decision-making. They clarify what should be evaluated and provide the foundation for distinguishing expected variation from menstrual cycle changes that should prompt evaluation, referral, or planned follow-up within primary care.

## Applying the framework across the lifespan

The clinical meaning of menstrual cycle change varies across the lifespan, but the interpretive task remains consistent: to determine whether a pattern represents expected variation, adaptive physiological change, or a signal requiring evaluation for Primary Ovarian Insufficiency or another underlying condition. Applying the framework across developmental stages requires attention to age-specific context while maintaining predefined criteria for action, including persistence, progression, absence of an explanatory context, and associated clinical features.

After menarche, menstrual variability is common as hypothalamic–pituitary–ovarian axis maturation proceeds. However, developmental variability should not be used to dismiss clearly abnormal patterns. An interval of more than 90 days between menses is abnormal after menarche and warrants evaluation, particularly when the pattern is persistent, recurrent, progressive, or associated with symptoms suggesting estradiol deficiency or broader endocrine dysfunction (7, 8). In this age group, structured interpretation helps avoid premature reassurance and supports timely consideration of Primary Ovarian Insufficiency among other endocrine, genetic, or gynecologic causes, including adolescent gynecologic conditions such as dysmenorrhea and endometriosis when symptoms are suggestive (30).

In early and mid-adulthood, menstrual cycle patterns are typically more stable, making new changes in cycle frequency, regularity, duration, or amenorrhea more clinically significant. In this setting, persistent or progressive cycle disruption should prompt evaluation for pregnancy, endocrine disorders, functional hypothalamic suppression, polycystic ovary syndrome, medication effects, structural pathology, and Primary Ovarian Insufficiency when clinically appropriate (28, 31–33). When menstrual disturbance is accompanied by vasomotor symptoms, sleep disturbance, dyspareunia, genitourinary symptoms, or other features suggestive of estradiol deficiency, Primary Ovarian Insufficiency should be considered, and timely referral should be pursued when initial evaluation supports concern.

In midlife, increasing variability in menstrual cycle length and bleeding patterns may reflect the menopausal transition, but age-related transition does not eliminate the need for clinical interpretation. The Stages of Reproductive Aging Workshop framework describes menstrual cycle changes as part of reproductive aging, and contemporary menopause guidance emphasizes evaluation when bleeding patterns are atypical, symptoms are clinically significant, or other pathology is suspected (34, 35). In this context, the framework supports distinguishing the expected reproductive transition from patterns requiring assessment, including persistent abnormal bleeding, marked symptom burden, genitourinary symptoms associated with estradiol deficiency, or features suggesting coexisting endocrine, structural, or systemic disease (35, 36).

Across these stages, follow-up and longitudinal care serve related but distinct functions. Follow-up refers to planned reassessment of a defined menstrual signal within a specified clinical interval when immediate referral is not required, but diagnostic vigilance remains necessary. Longitudinal care refers to the continuing clinician–patient relationship through which patterns can be recognized across repeated encounters, interpreted in context, and coordinated with specialty care when needed. This distinction is central to applying the framework safely: mild or contextually explained changes may be followed with planned reassessment, whereas persistent, progressive, unexplained, or clinically concerning patterns should prompt evaluation or referral.

Applied across the lifespan, the framework does not treat menstrual cycle patterns as diagnostic in themselves. Rather, it uses age-specific context, predefined criteria, and longitudinal observation to support timely clinical decisions. In this way, Primary Ovarian Insufficiency remains the sentinel condition, while the same structured reasoning helps primary care clinicians avoid both premature reassurance and unnecessary escalation.

## Integrating structured interpretation into primary care workflows

Primary care is the setting in which early menstrual signals are most likely to be observed, documented, and followed over time. For Primary Ovarian Insufficiency, this matters because delayed recognition often occurs despite repeated clinical encounters (1–3). At the same time, established guidance has long emphasized that amenorrhea and clinically significant menstrual disturbance warrant timely evaluation (7, 23, 24, 28, 32). The challenge, therefore, is not simply awareness but implementation within the constraints of routine care.

Workflow integration should begin with information already available in the clinical encounter. Because time constraints limit what can be added to primary care visits (21), the framework relies on a small set of practical questions: Is the menstrual pattern new or changing? Is it persistent or progressive? Is there an apparent explanation, such as pregnancy, medication, systemic illness, weight change, obesity-related endocrine effects, or functional hypothalamic suppression (31, 37)? Are there associated symptoms suggesting estradiol deficiency or broader endocrine dysfunction? These questions turn menstrual history into a triage process rather than a separate task.

**Figure 2:**
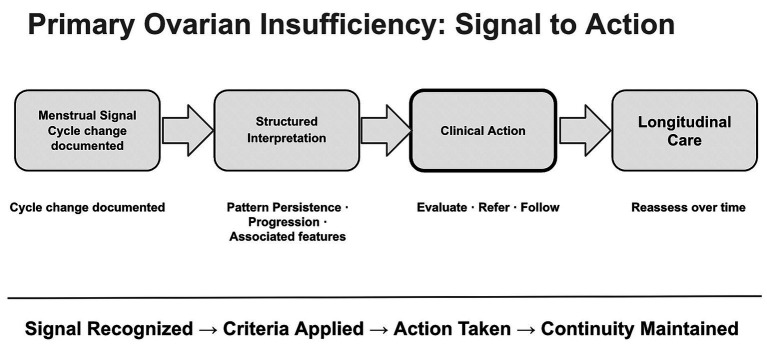
Primary ovarian insufficiency: from menstrual signal to clinical action. Menstrual cycle changes are interpreted as clinical signals using predefined criteria, including pattern, persistence, progression, and associated clinical features. These criteria guide decisions to evaluate, refer, or follow with planned reassessment. Longitudinal care supports recognition of change over time and helps prevent early signals from being lost between encounters.

When a pattern is persistent, progressive, unexplained, or clinically concerning, diagnostic evaluation is warranted. Initial assessment may include pregnancy testing when relevant, review of medications and systemic illness, assessment for functional hypothalamic suppression and other endocrine conditions, and targeted laboratory testing guided by the clinical presentation (28, 31, 32). Referral is appropriate when findings suggest Primary Ovarian Insufficiency, when amenorrhea or marked cycle disruption persists, when symptoms of estradiol deficiency are present, when initial testing is abnormal, or when diagnostic uncertainty remains (23, 24). The resulting clinical pathway is summarized in Figure 2.

Follow-up should be explicit rather than open-ended. In this framework, follow-up means planned reassessment of a defined menstrual concern within a specified clinical interval. Longitudinal care is broader: it refers to the continuing clinician–patient relationship that allows patterns, symptoms, test results, and context to be interpreted across repeated encounters. This distinction is important because continuity of care supports recognition, coordination, and improved outcomes over time (16, 17). A mild, contextually explained change may be followed with reassessment; a persistent or unexplained pattern should not be allowed to drift.

Documentation is the practical mechanism that makes this possible. Recording cycle frequency, regularity, duration, volume, amenorrhea, persistence, progression, and associated symptoms creates comparable observation points across visits. This also incorporates a methodological point: standardized timing becomes consistent documentation across encounters; predefined criteria become explicit thresholds for evaluation or referral; and bias reduction means avoiding premature attribution of menstrual disturbance to adolescence, stress, weight change, or midlife transition without checking whether concerning criteria are present.

Patient preparation can further improve the efficiency of care. A Primary Ovarian Insufficiency Readiness initiative could help individuals recognize secondary amenorrhea and related concerns, organize their history, and communicate more effectively during clinical encounters (38). Used alongside clinician-facing interpretation, patient-facing preparation may improve the alignment between what patients report and how clinicians respond.

Integrated in this way, the framework does not require a separate clinical pathway. It uses menstrual information already present in primary care, applies explicit criteria to determine the next step, and relies on continuity to prevent early signals from being lost between visits.

## Limitations of a pattern-based clinical framework

A pattern-based framework can strengthen recognition of menstrual cycle changes, but it cannot diagnose Primary Ovarian Insufficiency or any other condition on its own. Amenorrhea and other menstrual disturbances require clinical evaluation because the differential diagnosis is broad, including pregnancy, functional hypothalamic suppression, polycystic ovary syndrome, thyroid disease, hyperprolactinemia, medication effects, systemic illness, structural pathology, and Primary Ovarian Insufficiency (28, 31–33). Thus, menstrual pattern recognition should be understood as a triage aid, not a diagnostic endpoint.

This limitation is especially important because the same menstrual pattern can reflect different mechanisms depending on age and context. After menarche, cycle variability may accompany hypothalamic–pituitary–ovarian axis maturation, but an interval of more than 90 days between menses remains abnormal and warrants assessment (7, 8). In midlife, cycle length variability may reflect reproductive aging, but atypical bleeding or clinically significant symptoms still require evaluation rather than automatic attribution to transition alone (34, 35).

The framework also depends on the quality and continuity of clinical information. Incomplete recall, inconsistent documentation, and limited access to prior records reduce the reliability of pattern interpretation. Continuity of care can mitigate this limitation by allowing changes to be recognized across repeated encounters and interpreted in the clinical context (16, 17). When care is fragmented, early menstrual signals may be documented without being connected into a meaningful pattern.

A further limitation is the risk of both over-interpretation and under-recognition. Over-interpretation may lead to unnecessary testing or referral when a change is transient and clinically explained. Under-recognition may occur when persistent or progressive changes are normalized as part of adolescence, stress, body weight changes, or a midlife transition. The framework attempts to reduce both risks through predefined criteria, explicit reassessment, and attention to associated clinical features, but its usefulness depends on clinical judgment and follow-through.

Finally, this Perspective proposes a clinical reasoning framework rather than a validated diagnostic instrument. Future research should assess whether structured menstrual cycle interpretation reduces time to diagnosis, improves referral appropriateness, strengthens continuity of care, or improves patient-reported outcomes in Primary Ovarian Insufficiency and related presentations.

## Future directions: coordinated care and longitudinal data systems

Future work should test whether structured menstrual cycle interpretation improves care for Primary Ovarian Insufficiency in real-world settings. The rationale is strong: delayed recognition remains common despite repeated clinical encounters (1–3), and strong primary care systems are associated with improved population health outcomes (17). The next step is to move from a proposed clinical reasoning framework to implementation studies that assess time to diagnosis, referral quality, continuity of care, and patient-reported outcomes.

Primary Ovarian Insufficiency is well-suited to this kind of systems-level work because it is uncommon, clinically consequential, and most often preceded by menstrual disturbance. Longitudinal data systems could help characterize the interval between early menstrual changes and diagnosis, identify common points of delay, and support more consistent care pathways. Life course epidemiology provides a useful foundation for this approach because it emphasizes how health trajectories unfold over time rather than at a single clinical encounter (39, 40).

A centralized registry or natural history study could capture menstrual cycle patterns, associated symptoms, laboratory findings, diagnoses, referrals, treatments, and outcomes over time. Natural history studies are particularly important in rare diseases because they help define disease trajectories, support earlier signal detection, and provide infrastructure for future interventional research (41). For Primary Ovarian Insufficiency, this would allow menstrual cycle disturbances to be studied not only as a presenting symptom but also as part of a longitudinal clinical pathway.

Coordinated care models should also be evaluated. Within such a model, primary care would remain the point of first contact, while specialty care, patient navigation, and community-based support, including online peer-support spaces, would be organized around shared criteria for evaluation and referral. Continuity of care is central to this design because repeated encounters allow clinical signals to be recognized, interpreted, and acted upon over time (16, 17). Patient navigators may further reduce fragmentation by identifying barriers, coordinating referrals, supporting follow-up, and helping individuals move across primary care, diagnostic testing, and specialty services (43–45).

Patient-centered data ownership is another important direction. Patient-generated health data may improve continuity of care when individuals receive care from different clinicians or systems, but it also raises practical challenges related to data quality, privacy, security, and clinical usability (42). For menstrual cycle interpretation, patient-held records could help preserve longitudinal information that might otherwise be lost between visits, provided that such data are integrated into care in a clinically responsible way.

Together, these directions suggest a coordinated model in which menstrual cycle patterns are captured longitudinally, interpreted using predefined clinical criteria, and connected to timely evaluation and referral when needed. Primary Ovarian Insufficiency offers a practical starting point for developing and testing this model because the presenting signal is often available before diagnosis, the consequences of delay are clinically meaningful, and improvement can be measured through concrete outcomes.

## Conclusion

Primary Ovarian Insufficiency illustrates why menstrual cycle patterns should be interpreted as clinical signals rather than recorded as isolated history. Menstrual disturbance often precedes diagnosis, yet delays remain common despite repeated clinical encounters (1–3). This gap persists even though amenorrhea and clinically significant menstrual disturbance have long been recognized as indications for evaluation (7, 23, 24, 28, 32).

A structured approach can help close this gap by making explicit how menstrual patterns inform clinical reasoning. Cycle frequency, regularity, duration, volume, amenorrhea, persistence, progression, and associated symptoms provide practical information for determining whether a patient should be evaluated, referred, or followed with planned reassessment. These elements do not replace diagnostic evaluation; rather, they help primary care clinicians recognize when such evaluation is needed.

The value of this framework lies in its feasibility. Menstrual history is already part of routine care, and primary care offers continuity across time. When menstrual changes are consistently documented and interpreted according to predefined criteria, early signals are less likely to be missed between visits. This is especially important for Primary Ovarian Insufficiency, where earlier recognition may improve diagnostic timing, coordination of care, and attention to systemic health implications, including bone, cardiovascular, reproductive, and psychosocial health. By treating menstrual cycle patterns as actionable clinical signals rather than isolated historical details, primary care can support more timely evaluation, appropriate referral, and continuity of care for individuals at risk for Primary Ovarian Insufficiency.

## Data Availability

The original contributions presented in the study are included in the article/supplementary material, further inquiries can be directed to the corresponding author.
